# Hemoptysis Revealing Microscopic Polyangiitis: A Case Report

**DOI:** 10.7759/cureus.86377

**Published:** 2025-06-19

**Authors:** Fatima Zahra Elazizi, Dalal Zagaouch, Soumia Fdil, Khalid Bouti, Sanaa Hammi

**Affiliations:** 1 Pulmonology Department, Mohammed VI University Hospital, Tangier, MAR; 2 Laboratory of Life and Health Sciences, Faculty of Medicine and Pharmacy of Tangier, Abdelmalek Essaâdi University, Tangier, MAR

**Keywords:** alveolar hemorrhage, anca-associated vasculitis, anti-mpo antibodies, cyclophosphamide, microscopic polyangiitis, pulmonary-renal syndrome

## Abstract

Microscopic polyangiitis (MPA) is a rare, necrotizing vasculitis affecting small-caliber vessels, commonly associated with anti-neutrophil cytoplasmic antibodies (ANCA), particularly anti-myeloperoxidase (MPO). We report the case of a 63-year-old male with a medical history of chronic smoking and hypothyroidism, who presented with hemoptysis, worsening dyspnea, and constitutional symptoms. Imaging revealed bilateral alveolo-interstitial infiltrates indicative of lung involvement, along with a distal pulmonary embolism. Laboratory investigations found anemia, rapidly progressive renal impairment, and positive anti-MPO perinuclear anti-neutrophil cytoplasmic antibodies (p-ANCA) at high titers, supporting a diagnosis of MPA. The patient was treated with high-dose corticosteroids and cyclophosphamide, with the immunosuppressive therapy adjusted according to his renal function. This case highlights the importance of considering MPA in patients with unexplained pulmonary and renal manifestations, especially in the presence of constitutional symptoms and positive ANCA serology.

## Introduction

Microscopic polyangiitis (MPA) is a necrotizing systemic vasculitis that affects small-caliber vessels. It typically manifests through clinical features such as glomerulonephritis and/or intra-alveolar pulmonary hemorrhages, as well as the presence of anti-neutrophil cytoplasmic antibodies (ANCA). It is a rare disease with an estimated prevalence ranging from 0 to 66 cases per million inhabitants in Europe [1].

This autoimmune pathology manifests clinically through multi-systemic involvement. Rapidly progressing glomerulonephritis, which can lead to end-stage renal failure, and possibly lethal intra-alveolar hemorrhages are the most common and severe symptoms. Other organs may be affected, such as the skin (purpura), peripheral nervous system (multiple mononeuropathy), or joints. Cardiovascular and gastrointestinal manifestations are rare but possible. Diagnosis relies on the association of suggestive clinical signs, the presence of ANCA, and, ideally, histological confirmation showing pauci-immune necrotizing vasculitis [1,2]. 

Treatment involves an initial induction phase designed to induce complete remission (achieved in ≥80% of cases within three months), followed by a maintenance phase to prevent relapse that occurs in about one-third of patients [1]. Early diagnosis is essential for initiating treatment that can be both lifesaving and prevent organ damage, but the nonspecific nature of clinical features makes this diagnostically challenging.

## Case presentation

**Figure 1:**
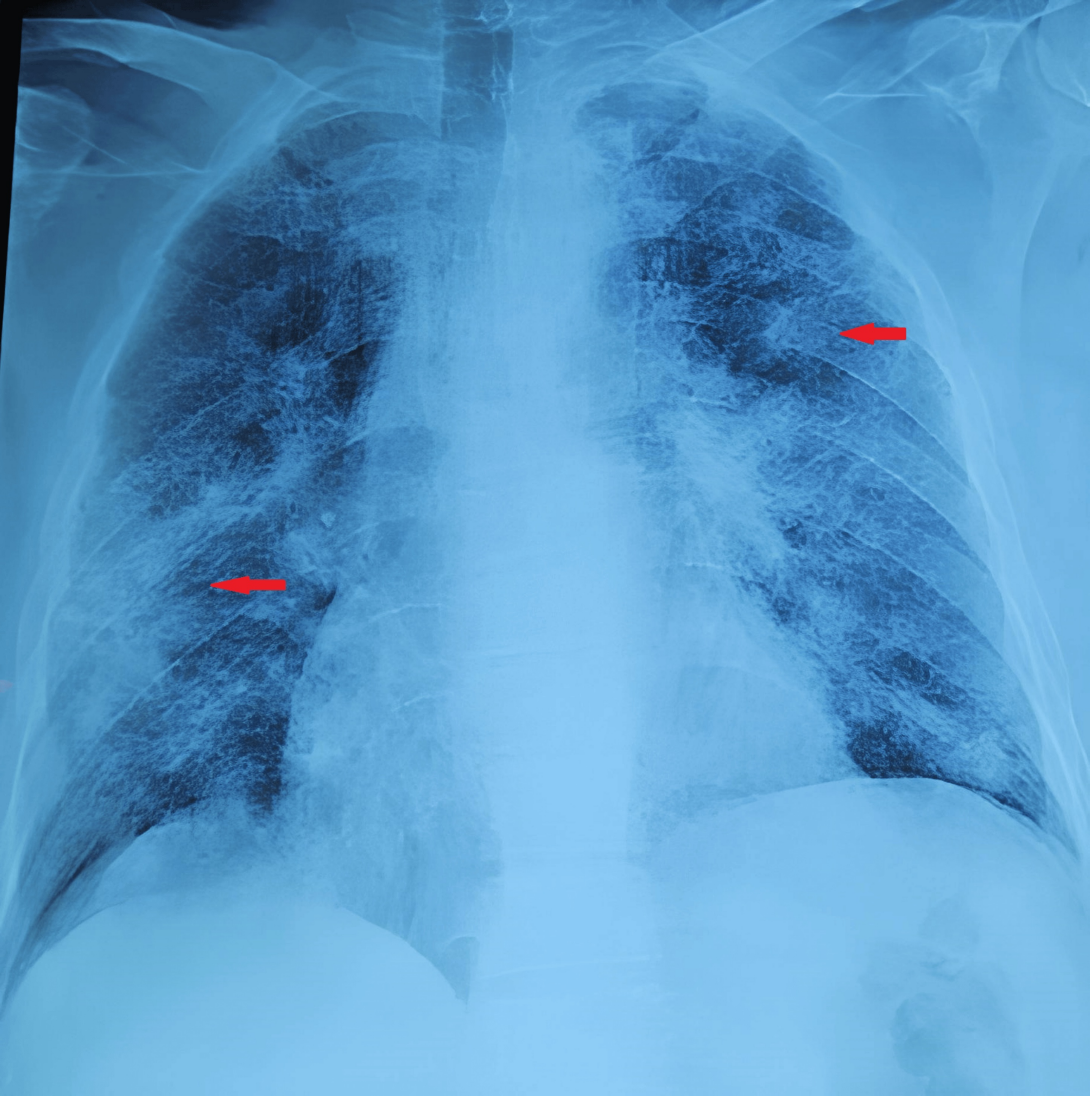
Chest X-ray Chest X-ray showing bilateral confluent alveolar and nodular opacities (arrow), forming an alveolo-interstitial syndrome.

We report the case of a 63-year-old patient with a history of chronic smoking (80 pack-years), bacteriologically confirmed pulmonary tuberculosis (TB) in 1988, and hypothyroidism treated with levothyroxine. The patient presented with low-volume hemoptysis associated with worsening chronic dyspnea progressing to stage 4 on the Modified Medical Research Council (mMRC) dyspnea scale. This was associated with constitutional symptoms including anorexia, asthenia, and a 6 kg weight loss over one month. Extrapulmonary manifestations included inflammatory polyarthralgia of large joints, an episode of epistaxis, crusting rhinitis, epigastric pain with one episode of vomiting, and constipation. The chest X-ray showed bilateral diffuse alveolar-interstitial opacities (Figure 1).

**Figure 2:**
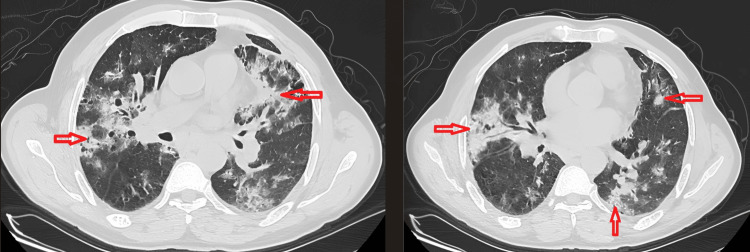
Chest CT scan Chest CT scan showing bilateral ground-glass opacities and alveolar consolidations with basal and middle lobe predominance, in the parenchymal window. CT, computed tomography

Given this radio-clinical presentation, we proceeded with D-dimer testing, which was elevated at 1091 ng/mL (reference: <500 ng/mL). Computed tomography (CT) angiography was subsequently performed and confirmed a distal left pulmonary embolism, along with multiple foci of pulmonary parenchymal consolidation and diffuse, bilateral ground-glass opacities, predominantly in the basal and middle lobar regions, with visible air bronchograms. The examination also revealed multiple bilateral, dense ground-glass pulmonary nodules and micronodules distributed in a peribronchovascular pattern (Figure 2).

Clinically at admission, vital signs revealed tachycardia (91 beats per minute) and tachypnea (25 breaths/min), with 94% oxygen saturation on room air. The patient appeared pale. Clinical examination revealed a decrease in vesicular murmurs and vocal vibrations bilaterally, more markedly on the right. The abdominal examination revealed diffuse abdominal tenderness, more pronounced in the epigastric and periumbilical areas.

Laboratory analysis revealed an inflammatory syndrome marked by an accelerated sedimentation rate of 130 mm and a significant rise in C-reactive protein (CRP) to 193 mg/L (reference: <6 mg/L) (Table 1). The blood count showed a regenerative microcytic hypochromic anemia, with a hemoglobin level of 6.6 g/dL (reference: 13-16 g/dL), while the hemolysis panel was negative. The patient also developed rapidly progressive acute renal failure, with an estimated glomerular filtration rate (eGFR) down to 18 mL/min, significant 24-hour proteinuria (3.04 g/24 h), and microscopic hematuria on urine cytobacteriological examination. The viral serologies for hepatitis B and C as well as HIV were negative. Given the patient's symptoms and prior TB history, we systematically investigated for active TB. Sputum GeneXpert, direct microscopy, and mycobacterial culture were all negative.

**Table 1:** Biological test results Bold values indicate abnormalities.

Parameter	Result	Reference Values
Leukocytes	9.20×10⁹/μL	4-10×10⁹/μL
Neutrophils	5.53×10⁹/μL (60.2%)	1.5-7.0×10⁹/μL (50-70%)
Lymphocytes	0.77×10⁹/μL (8.3%)	1-4×10⁹/μL (20-40%)
Monocytes	2.82×10⁹/μL (30.6%)	0.3-1×10⁹/μL (3-12%)
Eosinophils	0,03×10⁹/μL (0,3%)	0.1-0.4×10⁹/μL (0.5-5%)
Basophils	0.05×10⁹/μL (0.5%)	<0.1×10⁹/μL (0-1%)
Red Blood Cells (RBCs)	2.54×10⁶/μL	4.5-5.8×10⁶/μL
Hemoglobin (HGB)	6.6 g/dL	13-16 g/dL
Hematocrit (HCT)	20.9%	37-47%
Mean Corpuscular Volume (MCV)	82.5 fL	80-100 fL
Mean Corpuscular Hemoglobin (MCH)	26.0 pg	27-32 pg
Mean Corpuscular Hemoglobin Concentration (MCHC)	31.4 g/dL	32-36 g/dL
Red Cell Distribution Width (RDW-CV)	14.9%	11.5-14.5%
Red Cell Distribution Width (RDW-SD)	52 fL	-
Platelets (PLTs)	234×10⁹/μL	150-450×10⁹/μL
Mean Platelet Volume (MPV)	10.0 fL	6.5-12 fL
Platelet Distribution Width (PDW)	16.8	9-17
C-Reactive Protein (CRP)	193 mg/L	<6 mg/L
Erythrocyte Sedimentation Rate (ESR/VS)	130 mm	<20 mm (variable by age and sex)
Urea	0.45 g/L	0.06-0.36 g/L
Serum Creatinine	22.7 mg/L	7-13 mg/L
Estimated Glomerular Filtration Rate (eGFR, MDRD method)	18 mL/min	>90 mL/min
24 h Proteinuria	3.04 g/24 h	<0.15 g/24h
D-dimer	1091 ng/mL	<500 ng/mL
Perinuclear anti-neutrophil cytoplasmic antibodies (p-ANCA (Ac anti-MPO))	Positive 1/40 (123 IU/mL)	<1/10 negative, >1/10 positive (0-5)

Faced with this clinical presentation suggestive of a pneumo-renal syndrome, and in the context of suspected ANCA-associated vasculitis, we completed the biological assessment with testing for p-ANCA, which were positive for anti-myeloperoxidase (MPO) antibodies at a titer of 1/40, with a significantly elevated concentration of 123 IU/mL (reference: 0-5 negative), while antinuclear antibodies and anti-glomerular basement membrane antibodies were negative. According to ACR/EULAR 2022 classification criteria, the score was six points, supporting the diagnosis of MPA.

To further support the diagnosis, we completed the investigation with biopsies of the nasal cavities. Macroscopically, the nasal mucosa appeared healthy, and biopsies were taken from the lateral walls of the right and left nasal cavities. The histopathological examination revealed low-grade dysplasia of the nasal mucosa.

Following diagnosis confirmation, the patient received induction treatment with corticosteroid boluses of 15 mg/kg/day of methylprednisolone (1 g/day for three days), followed by a maintenance regimen of prednisolone 60 mg per day with a reduction schedule. A cyclophosphamide "Endoxan" bolus of 800 mg was initiated four days after the corticosteroid boluses, with a reduced dose of 10 mg/kg according to the renal insufficiency protocol (creatinine clearance <30 mL/min), in association with Uromitexan "Mesna." Six cyclophosphamide boluses were to be administered until March 2025.

**Figure 3:**
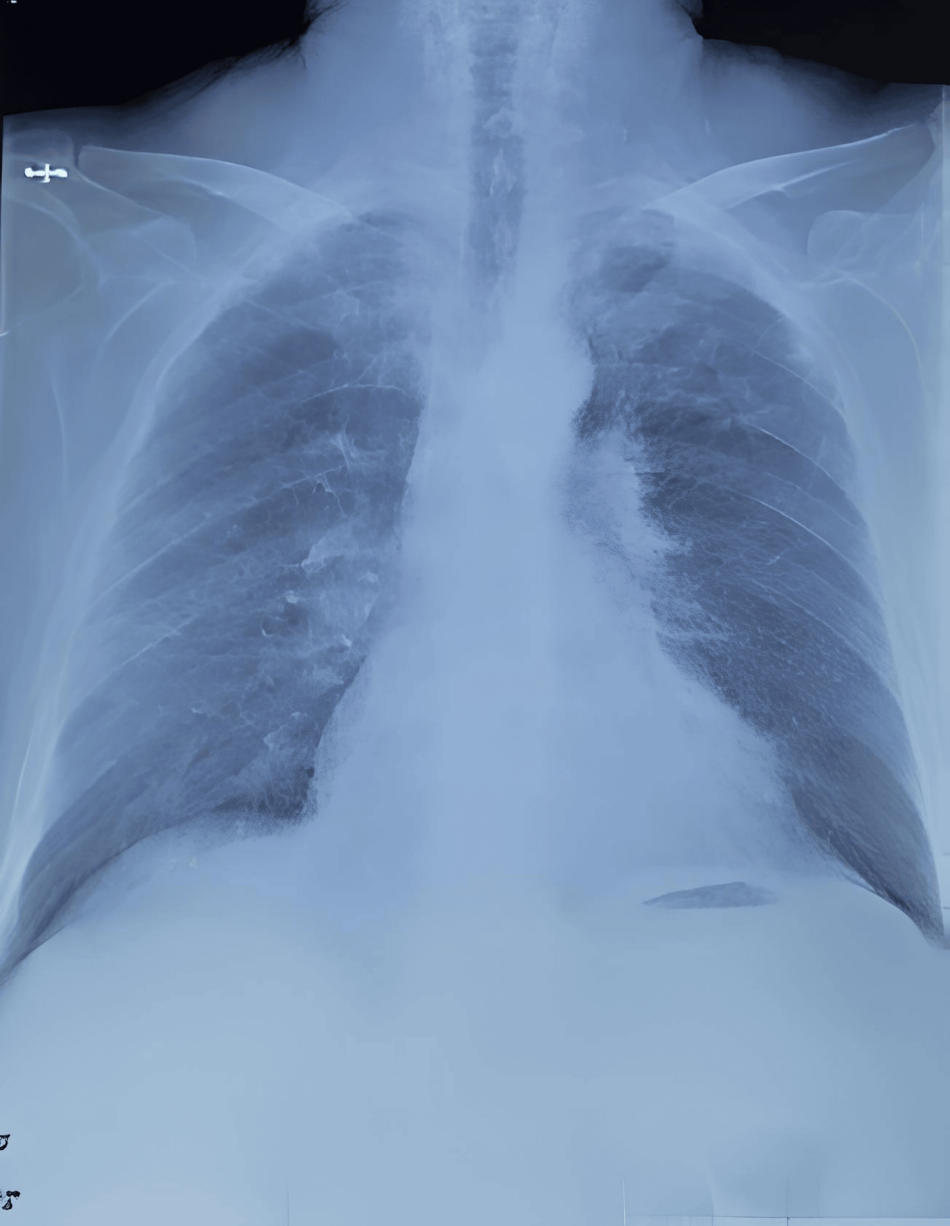
Chest X-ray Chest X-ray showing resolution of bilateral alveolar opacities and nodules.

The patient's clinical evolution was characterized by marked improvement, with complete resolution of hemoptysis and significant reduction in dyspnea. Following the third cyclophosphamide cycle, renal function showed improvement with an eGFR of 51 mL/min (vs. 18mL/min). The radiological assessment demonstrated the clearing of pulmonary lesions with decreased nodular opacities (Figure 3).

## Discussion

MPA is a systemic necrotizing vasculitis affecting small vessels, with the presence of ANCA in 75% of cases. It is usually associated with MPO-ANCA, although anti-proteinase 3 (PR3) ANCAs may also be observed [1,2]. The disease often causes extracapillary necrotizing glomerulonephritis, associated with the involvement of the skin, muscles, joints, digestive tract, and/or lungs (e.g., alveolar hemorrhage) [3].

Inaugural general symptoms, such as fever and malaise, may precede organ-specific involvement weeks or months before a more pronounced form of the disease. Our patient presented prodromal symptoms characterized by asthenia, anorexia, epistaxis with crusty rhinitis, and inflammatory polyarthralgia affecting the large joints.

Alveolar hemorrhage, due to pulmonary capillaritis, occurs in 12% to 29% of MPAs and can be revealing. Hemoptysis can be moderate or massive and is responsible for anemia, respiratory distress, or even shock [4]. Our patient had hemoptoic sputum, with progressive worsening and low-volume hemoptysis. When alveolar hemorrhage is associated with glomerulonephritis, it constitutes a pneumo-renal syndrome.

Renal involvement is one of the major characteristics of MPA. It occurs insidiously in three-quarters of patients, manifesting as a pauci-immune progressive glomerulonephritis.

Clinically, the progression is characterized by a rapidly progressive decline in renal function, preceding the appearance of hematuria and glomerular proteinuria [4]. In our case, the patient initially had normal renal function, which deteriorated within a month, with creatinine clearance falling to 18 mL/min. The kidney biopsy, when performed, can reveal the coexistence of acute glomerulonephritis lesions and glomerular scars. In our patient, performing the kidney biopsy presented significant risks. He was on curative-dose anticoagulant treatment, initially prescribed for pulmonary embolism, which significantly increased the risk of hemorrhagic complications. The main risks included bleeding at the puncture site and perirenal hematoma. These risks required a precise evaluation of the benefit-risk ratio before proceeding with the diagnostic procedure.

In most patients, a marked inflammatory syndrome is observed. Anemia, often significant, can result from two distinct mechanisms: inflammation or alveolar hemorrhage [1]. Our patient presented severe anemia at 6.6 g/dL, regenerative, with a negative hemolysis evaluation. In the context of repetitive low-abundance hemoptysis, this anemia was probably related to a hemorrhagic mechanism. The patient was transfused with three units of packed red blood cells, which raised his hemoglobin level to 7.9 g/dL.

Anti-MPO antibodies are a variety of ANCA. They are characteristic of certain systemic vasculitis, particularly MPA [3]. In rare situations, less than 5% of cases, cytoplasmic anti-neutrophil cytoplasmic antibodies (c-ANCA) anti-PR3 and p-ANCA anti-MPO antibodies may coexist, or c-ANCA antibodies alone may be present [1]. The ANCA screening for our patient was positive by indirect immunofluorescence, with p-ANCA anti-MPO specificity confirmed by ELISA and a significantly elevated titer of 123 IU/mL. The diagnostic score, calculated according to ACR/EULAR 2022 classification criteria, was six points positive, confirming the diagnosis of MPA [5].

The radiographic features of MPA are characterized by bilateral alveolar consolidations, indicating the presence of alveolar hemorrhage. Pleural effusion is observed in about 15% of cases, while pulmonary edema occurs in 5% [6]. Our patient's thoracic CT angiography revealed diffuse, non-systematized, ground-glass alveolar opacities, bilateral with a predominance in the basal and middle lobes. The presence of multiple nodules, some of which were excavated, wrongly led us to suspect granulomatosis with polyangiitis (formerly Wegener's disease).

The therapeutic strategy for systemic vasculitis relies on two essential phases. The first induction phase aims to achieve complete remission. This is followed by maintenance treatment designed to prevent any relapses. Corticosteroid therapy is the fundamental pillar of the therapeutic protocol. In forms considered severe, this approach is complemented by a combination of immunosuppressive agents [7-9].

The first-line induction treatment is cyclophosphamide or rituximab. RAVE and RITUXVAS studies demonstrated the non-inferiority of rituximab compared to cyclophosphamide [7,8]. In case of relapse, rituximab is particularly promising, with a significantly higher remission rate, reaching 67% compared to 42% according to the study by Stone et al. [7]. Plasma exchanges are reserved for the most critical clinical situations, especially in the presence of alveolar hemorrhage or persistent deterioration of renal function despite optimal immunosuppressive treatment [9].

The MAINRITSAN and RITAZAREM clinical trials demonstrated the superiority of rituximab compared to azathioprine in the maintenance treatment of ANCA-associated systemic necrotizing vasculitis [10,11]. The WEGENT study has also demonstrated that methotrexate is an equivalent alternative to azathioprine if renal function is compatible with its use [12].

The treatment regimen began with a 1 g bolus of corticosteroid therapy administered for three days, followed by prednisone 60 mg/day. A reduced dose bolus of cyclophosphamide at 10 mg/kg was then administered.

Our patient presented with severe vasculitis, characterized primarily by pulmonary and renal involvement. This clinical assessment justified the use of a therapeutic protocol combining cyclophosphamide and corticosteroid, followed by maintenance treatment with azathioprine [13].

## Conclusions

MPA remains a rare but potentially life-threatening systemic vasculitis characterized by its complex and polymorphic clinical presentation. Our experience underscores the critical importance of a multidisciplinary approach, with nephrology, pulmonology, rheumatology, and pathology collaborating seamlessly in both diagnosis and management. The treatment strategy, balancing aggressive immunosuppression during induction with careful monitoring for complications, followed by tailored maintenance therapy, highlights the necessity of personalized medicine in vasculitis care. This case contributes valuable insights to the evolving understanding of MPA and reinforces that early recognition, comprehensive evaluation, and coordinated care across specialties remain the cornerstones of optimizing outcomes in this challenging condition.
